# Linguistic experiential priors account for notation-dependent numerical representations

**DOI:** 10.1016/j.cognition.2026.106529

**Published:** 2026-04-01

**Authors:** Giorgia Anceresi, Krzysztof Franciszek Zdanowski, Marco Marelli, Avishai Henik, Luca Rinaldi

**Affiliations:** a Department of Brain and Behavioral Sciences, University of Pavia, Pavia, Italy; b Department of Psychology, University of Milano-Bicocca, Milano, Italy; c NeuroMI, Milan Center for Neuroscience, Milano, Italy; d Department of Psychology, Ben-Gurion University of the Negev, Beer-Sheva, Israel; e The Zelman Center for Brain Science, Ben-Gurion University of the Negev, Beer-Sheva, Israel; f Cognitive Psychology Unit, IRCCS Mondino Foundation, Pavia, Italy

**Keywords:** Numerical cognition, Distributional semantic models, Number notation, Distance effect

## Abstract

The question whether the representation of numbers is abstract and notation-independent still sparks a fervent debate in the numerical cognition field. Here, we employed distributional semantic models to quantify the distributional history of Arabic digits and number words in natural language (i.e., from large-scale written corpora). In a first computational experiment, we demonstrated that the distributional history of numbers in language reflected distance effect-like relationships for both numerical notations. Next, in a second behavioral experiment, we used these specific distributional histories as notation-dependent experiential priors to predict participants’ performance in comparison tasks employing both Arabic digits and number words. We observed that the linguistic experiential priors are not only constituting better models for behavioral performance than the real numerical distance, but we also found evidence for (partial) notation-dependent behavior. That is, our results showed that notation-dependent linguistic priors accounted for specific notation-dependent behaviors. These observations suggest that the specific distributional pattern of Arabic digits and number words in natural language reflect the way humans represent numbers, thus supporting an experiential-driven representation.

## 1 Introduction

Unravelling the neurocognitive mechanisms behind everyday quantity processing, as when comparing the number of items in two or more sets, has been a persistent goal in numerical cognition research. Notwithstanding the considerable progress in this field, there is still no consensus about whether numerical information is processed and represented abstractly. Namely, whether the same neurocognitive system would support quantity representations in a notation-independent and abstract fashion, or whether distinct systems would be involved depending on the input notation (e.g., Arabic digits vs. number words). Seminal theories, including the *triple-code model* proposed by Dehaene and colleagues (1992), suggested that while digits and number words would be initially represented in their respective codes, they would be then rapidly and automatically transformed into a common, abstract magnitude code. Along this line, prominent perspectives suggest the existence of an evolutionary old Approximate Number System (ANS) shared by many species and designed to process all varieties of non-symbolic magnitude (e.g., size, weight, numerosity) (Cantlon, Platt, & Brannon, 2009; Piazza, 2010). A hallmark of this system is ratio-dependent discrimination: the ability to discriminate two quantities depends on the ratio between their magnitudes, in accordance with Weber’s law (Cantlon et al., 2009; Leibovich et al., 2013). For example, performance in numerical comparison tasks typically declines as the numerical ratio (i.e., smaller set divided by larger set) approaches 1. Building on this theoretical framework, symbolic representations of number have been suggested to emerge later in development through mapping onto these pre-existing non-symbolic magnitude representations. The view of notation-independent representation of digits and number words has been supported by several lines of evidence (Dehaene & Akhavein, 1995; Libertus, Woldorff, & Brannon, 2007; McCloskey, 1992; McCloskey & Schubert, 2014). This includes findings from numerical comparison tasks, in which participants are asked to judge which among two numerals is larger or smaller (Aiken & Williams, 1968; Moyer & Landauer, 1967). Studies using these tasks have indeed shown that participants’ performance is similar across different notations and formats (e.g., Libertus et al., 2007).

When performing a number comparison task, accuracy and reaction times are systematically affected by the numerical distance between the quantities represented by the Arabic digits and number words. That is, the larger the numerical distance, the faster and more accurate the judgments, a phenomenon known as the distance effect (Henik & Tzelgov, 1982; Tzelgov et al., 1992). For example, participants will be slower and less accurate with comparison of 8 vs. 9 (distance equal to 1) than with 2 vs. 9 (distance of 7). Notably, the behavioral distance effect has been observed with different notations, and this has been taken as evidence for a notation-independent representation of numbers (Dehaene, Bossini, & Giraux, 1993). Similar results were reported in semantic priming tasks, with comparable priming semantic effects within and between notations (Naccache & Dehaene, 2001; Reynvoet, Brysbaert, & Fias, 2002). This view has also been substantiated by a large body of neuroimaging research showing that the intraparietal sulcus represents numbers irrespective of the notation used, be it symbolic (e.g., number words) or non-symbolic (e.g., dot patterns) (Dehaene, 1996; Eger, Sterzer, Russ, Giraud, & Kleinschmidt, 2003; Piazza, Izard, Pinel, Le Bihan, & Dehaene, 2004; Pinel, Dehaene, Rivière, & LeBihan, 2001).

Nonetheless, alternative perspectives supporting the hypothesis of separate notation-dependent processes are not missing. For instance, the *encoding complex hypothesis* assumes that number processing is mediated by modality-specific processes and not by an abstract code (Campbell, 1994). That is, the processing of Arabic digits and number words is thought to involve distinct representational systems that interact dynamically depending on task demands and context. This view is supported at the behavioral level by studies reporting different distance effects across notations (Bernardo, 2001; Campbell & Epp, 2004; Kadosh et al., 2008; Cohen Kadosh, 2008; see also Krajcsi, Lengyel, & Kojouharova, 2018). For example, Kadosh et al. (2008) found that when using a short response–stimulus interval (i.e., 200 ms between a participant’s response and the onset of the next stimulus), the distance effect was smaller for number words than for Arabic digits, suggesting an initial, automatic stage of processing involving notation-dependent representations. Further evidence emerges when considering bilingual individuals: Bernardo (2001) showed that Filipino–English bilinguals solved simple addition equations more quickly and accurately when problems were presented in digit format or in the language in which they had learned arithmetic (i.e., English), rather than in their first language (i.e., Filipino). This suggests that linguistic experience modulates access to numerical representations, supporting the view that symbolic number processing is shaped by notation- and language-specific factors. At the neural level, this view is supported by evidence of notation-specific differences in numerical processing in the parietal lobes (Kaas et al., 2007; see also Cao, Li, & Li, 2010).

Within this context, one of the current key theoretical frameworks is the Discrete Semantic Systems (DSS) model (Krajcsi et al., 2018; Krajcsi, Lengyel, & Kojouharova, 2016). According to this account, while non-symbolic number processing would be supported by the ANS, symbolic number processing relies on a semantic network of discrete nodes, akin to the mental lexicon. In this network, numbers are stored as individual units, and the strength of their associative links reflects their semantic proximity. Consequently, the numerical distance effect observed in symbolic comparison tasks can be explained by variations in associative strength between nodes. Within the DSS model, these associative relations are assumed to emerge as a product of associative learning mechanisms that integrate environmental statistics over time. For instance, Krajcsi and Kojouharova (2017) showed that the symbolic distance effect can arise from associations learned contingently during the experiment. In particular, participants were taught arbitrary, unfamiliar symbols corresponding to the values 1–3 and 7–9, while the intermediate values (4–6) were never introduced. Critically, comparisons such as “3 vs. 7” – whose numerical distance is 4 – were performed as quickly and accurately as comparisons between consecutively learned symbols, effectively behaving as if their distance were 1. These findings indicate that participants’ comparison performance was strongly shaped by recently acquired statistical information rather than by canonical values, thus reflecting the conditional probability structure of the symbolic representations.

The DSS model also accounts for another robust behavioral phenomenon, the numerical size effect – the tendency for smaller numbers to be processed more efficiently than larger ones – by attributing it to differences in symbol frequency rather than to analog magnitude ratios. This framework therefore provides an alternative explanation of distance and size effects without invoking an abstract numerical representation.

In light of the different theoretical debates discussed so far, here we aimed to shed light on this topic by taking a language processing perspective. Language learning can be indeed conceived to be a highly probabilistic process (Ramscar, 2019), with words having similar learning histories (in terms of distributional appearance over linguistic contexts) also tending to share similar meanings (i.e., the distributional hypothesis; Harris, 1954). Interestingly, advances in the field of computational linguistics allow for a convenient way to quantify words’ distributional histories and represent their meaning as high-dimensional numerical vectors (also known as word-embeddings) (Günther et al., 2019). These distributional semantic models (DSMs) are typically trained on large collections of texts that document natural language use.

Broadly, DSMs can be divided into count-based and prediction-based models (Baroni, Dinu, & Kruszewski, 2014). Count-based models, such as LSA (Landauer & Dumais, 1997) or HAL (Lund & Burgess, 1996), build semantic spaces by tracking how often words co-occur with other words or within linguistic units (e.g., documents, context windows). To derive semantic vectors, these raw co-occurrence counts are typically subjected to weighting schemes (e.g., Pointwise Mutual Information; PMI) and dimensionality reduction techniques (e.g., SVD). On the other hand, more modern prediction-based DSMs (e.g., *fastText*), are based on a neural network architecture in which nodes in the input and output layers represent words (Mikolov, Chen, Corrado, & Dean, 2013). The system learns to predict a target word on the basis of the lexical context in which it appears, or vice versa, depending on the specific model architecture used. For instance, in continuous bag of words (CBOW) models, the target word is being predicted by the linguistic context (i.e., the words it co-occurs with in the text). This is achieved by incrementally updating a set of weights which minimizes the difference between model predictions and observed data. The estimated set of weights will eventually capture linguistic behavior associated with a specific word in distributed terms.

These distributed representations, or vectors, can be quantitatively compared by measuring their proximity in a multidimensional space: similar words will occur in similar contexts, ending up being associated with vectors that are geometrically close (Landauer & Dumais, 1997), an assumption that is empirically supported (Baroni et al., 2014; Gatti et al., 2022; Mandera et al., 2017). Notably, prediction-based models are highly relevant for the study of human cognition because they have been linked to psychologically-grounded associative learning models (Günther et al., 2019; Mandera et al., 2017).

Interestingly, by employing DSMs, it has been demonstrated that the actual distance between number words can be in principle inferred solely from the distributional history of numbers in language (Rinaldi & Marelli, 2020). Notably, a recurrent critique posits that the information encoded in language may be only redundant and not constitute an independent source of knowledge (see Lupyan & Lewis, 2019 for further discussion). However, against this possibility, human performance in number comparison tasks has been shown to be better explained by linguistic similarity, as estimated by DSMs, than by real numerical distance: language thus appears to be an experiential source to represent the structural organization of number words (Rinaldi et al., 2022). Moreover, from a broader perspective, linguistic and perceptual information have been shown to provide not merely redundant, but rather complementary sources of information for the organization of semantic knowledge (Andrews, Vigliocco, & Vinson, 2009; Günther et al., 2022; Riordan & Jones, 2011).

Notably, by employing a DSM (Latent Semantic Analysis; Landauer & Dumais, 1997), Ren and Libertus (2024) recently showed that linguistic estimates predicted participants’ performance on a comparison task including both non-symbolic (i.e., dot patterns) and symbolic representations (i.e., number words). However, contrary to the authors’ hypothesis, linguistic estimates did not predict participants’ performance when Arabic digits were used. When interpreting this result, it is crucial to consider differences in models’ architecture used to estimate linguistic predictors. Indeed, traditional count-based models, such as LSA, build vector representations starting from co-occurrence patterns in a large corpus and applying dimensionality reduction techniques (Landauer & Dumais, 1997; Lenci, 2018). While effective for words with rich contexts, these models struggle with tokens that have limited or isolated usage (e.g., Mahmoud & Zrigui, 2021). Indeed, if a token appears in very few contexts (i.e., in only a handful of documents), its co-occurrence statistics are sparse and unreliable, leading to poorly defined or noisy embedding. This may be particularly relevant for less frequent numbers, but also for numbers appearing in very specific contexts. This may be the case for Arabic digits, which often occur in formulaic or specific contexts (e.g., dates, page numbers, lists), limiting in turn the variety of co-occurrences that LSA relies on to build meaningful vectors.

In addition to this, prediction-based models such as *fastText* (Joulin et al., 2016), contrary to count-based models including LSA, are considered to be mathematically consistent with psychologically plausible learning models (Günther et al., 2019; Mandera et al., 2017). Indeed, by relying on prediction processes embedded in neural network, it has also been shown that these models generally offer a better fit to humans’ behavioral data as compared to count-based ones (Mandera et al., 2017; Baroni et al., 2014).

Building upon these findings, we took advantage of DSMs (i.e., *fastText*; Joulin et al., 2016) allowing to induce meaning not only for number words but also from Arabic digits. This allowed us to obtain notation-dependent linguistic indices by extracting vector representations specific to either number words or Arabic digits. Notably, this approach aligns with the DSS model in assuming that symbolic numerical representations are shaped by statistical regularities in experience, and in positing representational units whose associative links reflect semantic proximity. However, whereas DSS remains largely agnostic about how such associations emerge, the present method leverages the distributional hypothesis (Harris, 1954) to offer a formal operationalization of specific experiential priors shaping semantic representation: namely, distributional patterns in natural language.

We therefore first asked whether the structural organization of numbers, as indexed by the distance effect, can be retrieved from the distributional regularities of number words and Arabic digits as extracted from natural language. In particular, we aimed at replicating and strengthening the evidence from works on number words (Rinaldi et al., 2022; Rinaldi & Marelli, 2020) and extending it to Arabic digits. Next, in a behavioral experiment, we asked participants to perform a number comparison task (i.e., with numbers presented in both notations) and predicted their performance from the notation-dependent linguistic indices, which thus served as linguistic experiential priors. We reasoned that linguistic information extracted from natural language reflects subtle but reliable notation-dependent differences in number representation. Thus, performance in the number comparison task should be better accounted for by the relative linguistic index than by numerical distance or by the linguistic index derived from the other notation. More precisely, we expected the linguistic index quantifying the distributional history of Arabic digits to emerge as the better predictor for performance in the digits comparison task, and vice versa for number words.

## 2 Experiment 1

In experiment 1 we explored whether the structural organization of numbers, as indexed by the distance effect, can be retrieved from the distributional regularities of number words and Arabic digits in natural language. This would replicate and strengthen previous evidence from works on number words (Rinaldi et al., 2022; Rinaldi & Marelli, 2020) and extend it to Arabic digits. Thus, we extracted vector representations for the number words and the Arabic digits from natural language. Then, we computed a vector distance (VD) index for each numerical pair. VDs were computed independently from number words or Arabic digits as to obtain notation-dependent linguistic indices (proxies for specific linguistic experiential priors with the two notations). Ultimately, we tested whether VDs for either number words or Arabic digits reflect the actual numerical distance (i.e., the absolute difference between the two to-be-compared numbers).

### 2.1 Methods

#### 2.1.1 Distributional semantic model

The DSM used here was *fastText* (Joulin et al., 2016), a widely distributed, prediction-based model belonging to the *word2vec* family (Mikolov, Chen, et al., 2013; Mikolov, Sutskever, Chen, Corrado, & Dean, 2013), trained on an Italian corpus of around 11 billion words (Grave, Bojanowski, Gupta, Joulin, & Mikolov, 2018). The model used to extract word vectors was trained using the Continuous Bag of Words (CBOW) method, an approach originally proposed by Mikolov and colleagues (2013), with position-weights across 300 dimensions, with character *n*-grams of length 5, a window of size 5 and 10 negatives. When using CBOW, the obtained vector dimensions capture the extent to which a target word is reliably predicted by the linguistic contexts in which it appears.

Crucially, with respect to traditional models, prediction-based models rely on cognitively plausible associative learning mechanisms and better account for human behavioral data (Günther et al., 2019; Mandera et al., 2017). Finally, *fastText* is based on the idea (originally proposed by Schutze, 1993; and realized by Bojanowski, Grave, Joulin, & Mikolov, 2017) to take into account sub-lexical information by computing word vectors as the sum of the semantic vectors for the character *n*-grams embedded in each word. This computational procedure yields embeddings that are highly convergent with standard *word2vec* representations for frequent words (Grave et al., 2018; Mandera et al., 2017), while also increasing the robustness and stability of vector representations across languages with large vocabularies and low frequency words (Bojanowski et al., 2017).

Importantly, the adoption of *fastText* in the present study reflects methodological considerations of performance, simplicity, and availability (see: https://fasttext.cc/docs/en/crawl-vectors.html). However, this methodological convenience does not reflect any theoretical assumptions about the cognitive encoding of numerical symbols (e.g., the use of subword information should not be interpreted as reflecting specific cognitive mechanisms underlying the processing of single-versus multi-power numbers).

From the semantic space, we therefore extracted vector representations for the number words and the Arabic digits included in this study. For each numerical pair, we computed a vector distance (VD) index based on the cosine of the angle formed by vectors representing the meanings of these words subtracted from 1. In particular, the lower the VD value, the more similar Arabic digits or number words used in language.

The full considered range consisted of Arabic digits and number words from 1 to 19. Due to the polysemous properties of Italian number words ‘uno’ (‘one’, also used as an indefinite article), ‘sei’ (‘six’, also used as a second-person form of the verb ‘to be’), and ‘venti’ (‘twenty’, also used to refer to ‘winds’) these numerals were excluded from the analysis in both notations. Other than that, for each remaining pair, we computed a VD as described above.

### 2.2 Data analysis and results

**Fig. 1:**
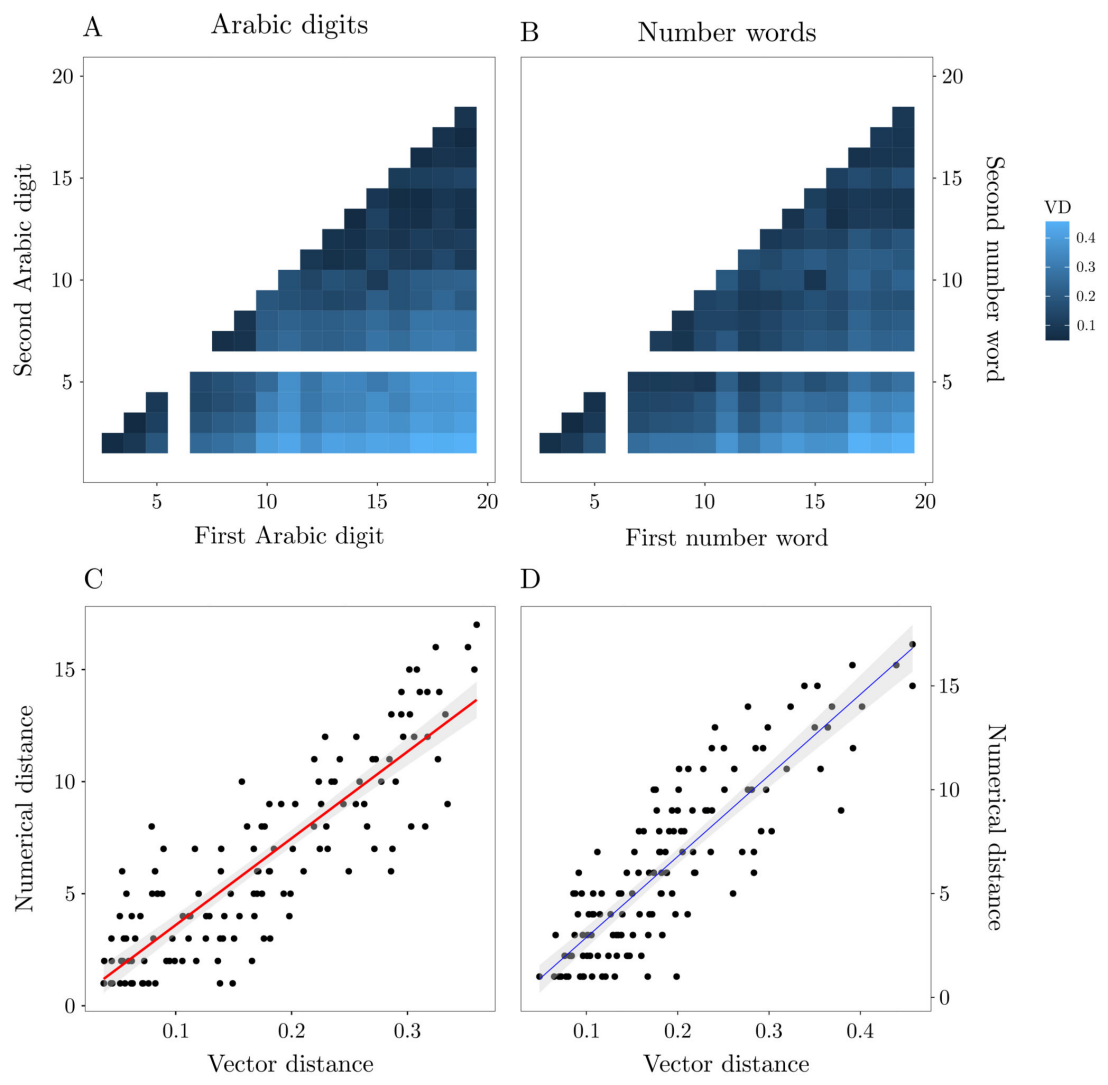
Heatmap for vector distance (VD) between pairs of Arabic digits (A) and numbers words (B). Darker colors indicate lower VD, that is, higher similarity in language usage. Note that the empty cells in the heatmaps identify the excluded numbers due to polysemic issues (i.e., 1 and 6). The positive correlations between numerical distance and VD for Arabic digits and number words are shown in (C) and (D), respectively.

A correlation analysis between real numerical distance and VD for Arabic digits showed a strong positive association (*r* = *0.86, p* < .*001*), see Fig. 1 (A and C). Similarly, for number words, the correlation analysis yielded a strong positive relationship (*r* = *0.85, p* < .*001*), see Fig. 1 (B and D). Then, we tested whether actual numerical distance is better captured by VD from Arabic digits or number words. Results showed that Arabic digits-based VD and number words-based VD did not differ significantly in their relationship with real numerical distance (*z* = − *0.25, p = .39*).^2^ Additionally, the correlation analyses between VDs of the two numerical notations also showed a strong positive relation between the two (*r* = *0.86, p* < .*001*).

Overall, both notation-based VDs were significantly correlated with their real numerical distances, suggesting that natural language word use follows real numerical distance, replicating previous results (Rinaldi & Marelli, 2020).

## 3 Experiment 2

In Experiment 2 we probed whether the data extracted from natural language can predict participants’ performance in a behavioral task. In particular, we asked participants to perform two number comparison tasks, one in which they had to compare number words and one in which they had to compare Arabic digits. Specifically, we explored whether the numerical distance effect can be better predicted from both experiential linguistic indices (VDs) compared to the real numerical distance. Moreover, to probe the hypothesis of notation dependency, we also tested whether experiential linguistic indices from the respective notation could predict performance more effectively. That is, we expected the linguistic indices from the specific notation (proxies for the relative experiential priors) to outperform the real numerical distance and to show notation specificity, also.

### 3.1 Methods

#### 3.1.1 Participants

We recruited 55 university students (39 females; age: *M* = 24.35 years, *SD* = 4.99), all native Italian speakers. They received academic credits for participation in the study. Out of the whole group, 51 were right-handed and 4 left-handed. Also, no learning disabilities possibly related to task performance (e.g., dyscalculia or other numerical processing disorders) were reported. The experiment was approved by the local ethics committee and informed consent was obtained from all participants before the experiment.

#### 3.1.2 Stimuli and procedure

The considered range of numbers we opted for in the current experiment was 1 to 9. We chose to present the full 1–9 range to ensure consistency in the stimulus set (and with respect to previous studies using this numerical range) but decided a priori to exclude specific items from the analysis. That is, as in Experiment 1, trials containing either 1 or 6 were excluded from the analysis in both notation sets due to polysemic issues. The focus was on two notations, hence two different comparison tasks — with Arabic digits and number words. We decided to not use double-digit numbers to avoid any confound due to the comparison between one and two-digit numbers in the Arabic comparison task, as two-digit numbers may be visually more salient and quickly identified as larger, even without a precise evaluation of their magnitude.

**Fig. 2:**
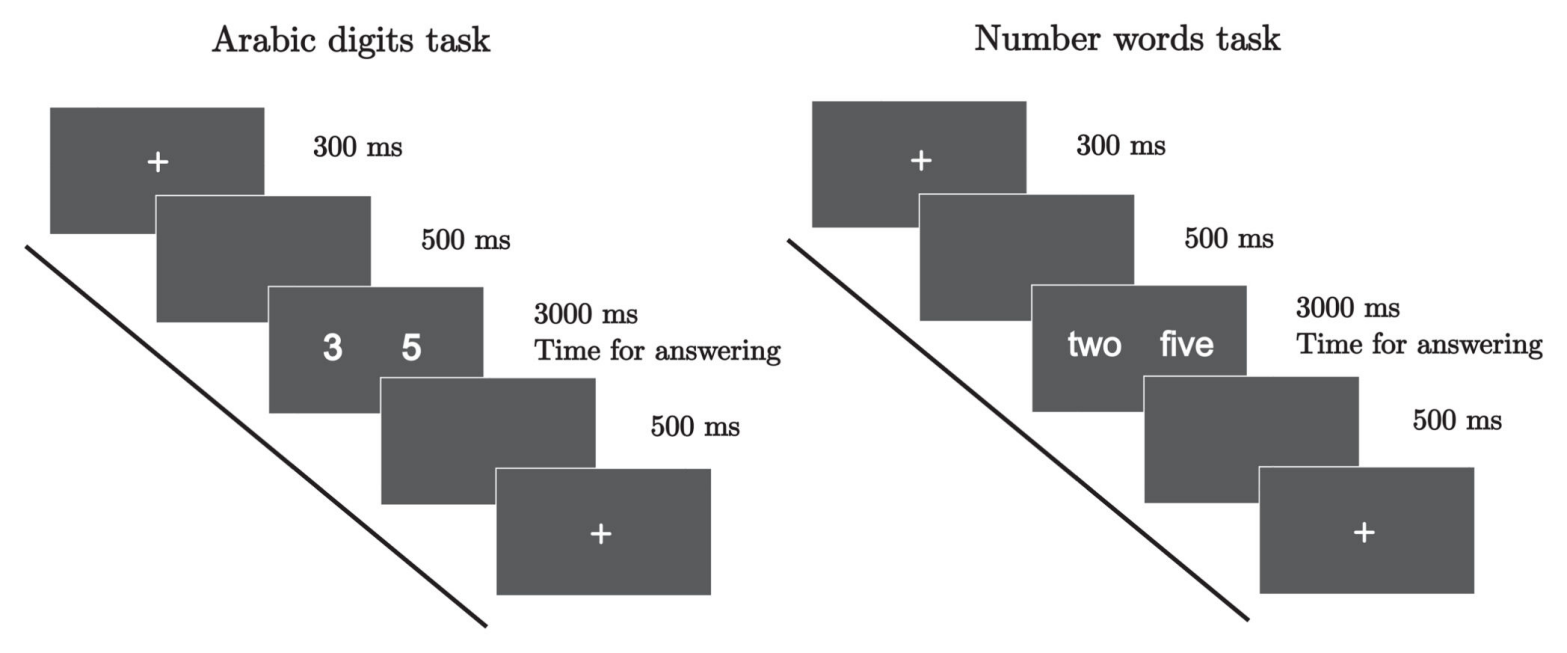
Trial structure for the Arabic digits (left) and number words (right) comparison tasks.

Both tasks consisted of presenting pairs of either Arabic digits or number words, placed at symmetrical distance from each other and from the centrally displayed fixation cross. All the trials started with a 300 ms fixation cross displayed centrally on the screen. The fixation cross was followed by 500 ms of blank screen and by the stimuli presentation, which lasted until participants’ response (for a maximum of 3000 ms). Participants were asked to respond as quickly and as accurately as possible by choosing the larger digit or number word with the relative keyboard keys (A if the larger digit appeared on the left side, L if it appeared on the right side). After the stimuli presentation, a blank screen was displayed for another 500 ms, ending the trial (Fig. 2).

Half of the participants were presented first with the Arabic digits task (*N* = 27), while the other half with the number words task (*N* = 28), to counterbalance any possible order effect.

All unique pair combinations of numbers 1 to 9 and Italian number words one (uno) to nine (nove) were used, accounting for their spatial layout of presentation (i.e., each stimulus appeared on the left and the right size of the screen; e.g., 2–5 and 5–2). Identical stimuli comparisons (e.g., 3–3) were omitted. This procedure resulted in 72 trials presented in each notation. Experiment 2 was conducted online via the Pavlovia portal using PsychoPy (Peirce & Mac Askill, 2018).

#### 3.1.3 Distributional semantic model

We used the same DSM as in Experiment 1. However, in this case, we extracted only VD from Arabic digits from 2 to 9 and from number words from *two* to *nine*, with the exclusion of 6 and *six*, respectively.

#### 3.1.4 Data analysis and preprocessing

For all analyses on reaction times (RTs), only observations in the range of 200 ms to 2000 ms were included, for a total of 4612 trials (with 7 trials excluded from the number words task for falling outside this range). This allowed us to focus on automatic intentional processing of stimuli only. All incorrect responses were also excluded, resulting in 2244 trials (97.1%) in the digits comparison task, and 2180 trials (94.7%) in the number words task. Using the *lme4* R package (Bates, Mächler, Bolker, & Walker, 2015), we estimated three different linear mixed models. Each model included log-transformed correct response times (RTs) as the dependent variable but differed in the predictor used: real numerical distance in the first model, VD from number words in the second, and VD from Arabic digits in the third. In each of the three models tested, we included a random intercept for subjects as well as for numerical pairs (treated irrespective of the placement of the two numbers on the screen; e.g., 2–4 stimuli pair equal to 4–2 stimuli pair).

After fitting the models, to exclude the impact of overly influential outliers, we checked whether the observed effects were significant also when removing data points based on a threshold of 2.5 *SD* standardized residual errors (model criticism; Baayen, Davidson, & Bates, 2008). Finally, the models were compared using the Akaike information criterion (AIC), which returns an estimation of the quality of the model in terms of information loss, with lower values indicating a better description of the data (Akaike, 1973). Crucially, AIC enables a principled comparison between alternatives models, quantifying their relative support rather than solely assessing the proportion of variance explained (Burnham & Anderson, 2002; West, Taylor, & Wu, 2012). In line with common practice, we focused on the ΔAIC relative to the best-fitting model, which can be translated into Akaike weights and evidence ratios to quantify the strength of support between competing models (Wagenmakers & Farrell, 2004). ΔAIC > 2 is generally regarded as indicative of evidence in favor of the lower-AIC model, as it would indicate that the model with lower AIC is 2.7 times more likely to minimize Kullback–Leibler information loss among the candidate models (Burnham & Anderson, 2002; Hilbe, 2011; Wagenmakers & Farrell, 2004). This adjudication approach also enables the comparison between the numerical-distance and linguistic-predictor models while avoiding collinearity issues and appropriately accounting for the inclusion of random effects (Bates et al., 2015; Burnham & Anderson, 2002). Results are presented for refitted models, while AIC weights are reported preceding the model criticism procedure.

Accuracy data was analyzed through generalized linear mixed models (GLMMs), comparing the three models with each other (each including one of the predictors, and using the same random intercepts employed for RTs).

## 4 Results

### 4.1 Reaction times

Participants’ mean reaction time in accurate trials in the Arabic digits task (excluding outliers not in 200 ms - 2000 ms range) was equal to 526.31 ms (*SD* = 138.75 ms), while in the number words task 830.49 ms (*SD* = 207.29 ms).

#### 4.1.1 Arabic digits task

**Fig. 3:**
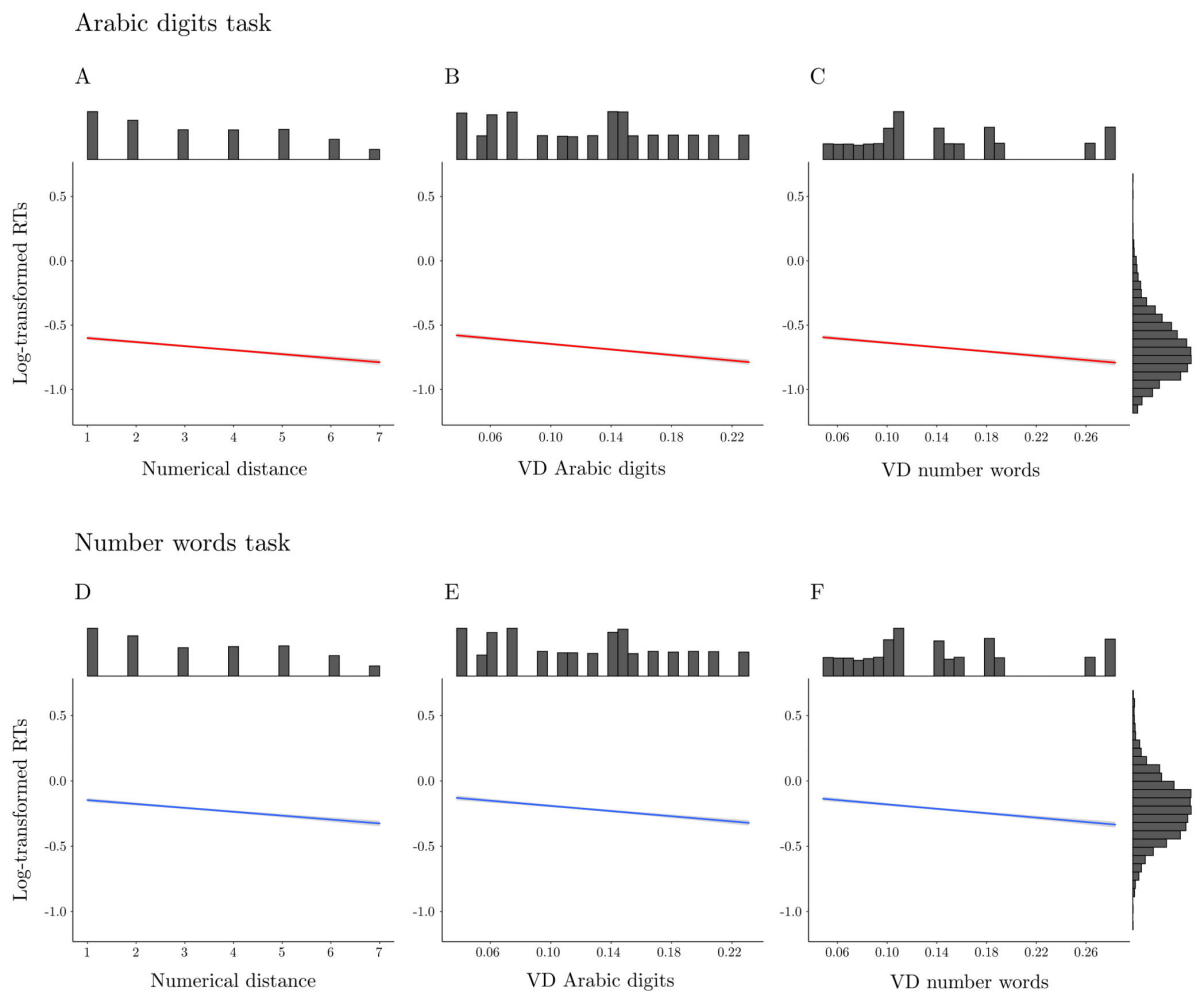
Plots illustrating results of the Arabic digits (upper panel) and the number words tasks (lower panel), with log-transformed RTs as dependent variable and numerical distance, VD derived from Arabic digits and from number words as predictors. Top and right-placed histograms indicate the distribution of variables, with marginal distribution for log-transformed RTs, and the distribution of numerical distance and VD predictors based on the respective variables.

In the Arabic digits task, we found a significant negative effect of numerical distance on RTs, *t*(*df* = 18.88343) = − 6.019, *p* < .001 (see Fig. 3A). A significant, negative effect was found also for the VD derived from Arabic digits; *t*(*df* = 18.81812) = −6.721, *p* < .001 (see Fig. 3B), as well as for the VD derived from number words; *t*(*df* = 18.82609) = −5.68, *p* < .001 (see Fig. 3C).

#### 4.1.2 Models comparison in the Arabic digits task

**Fig. 4:**
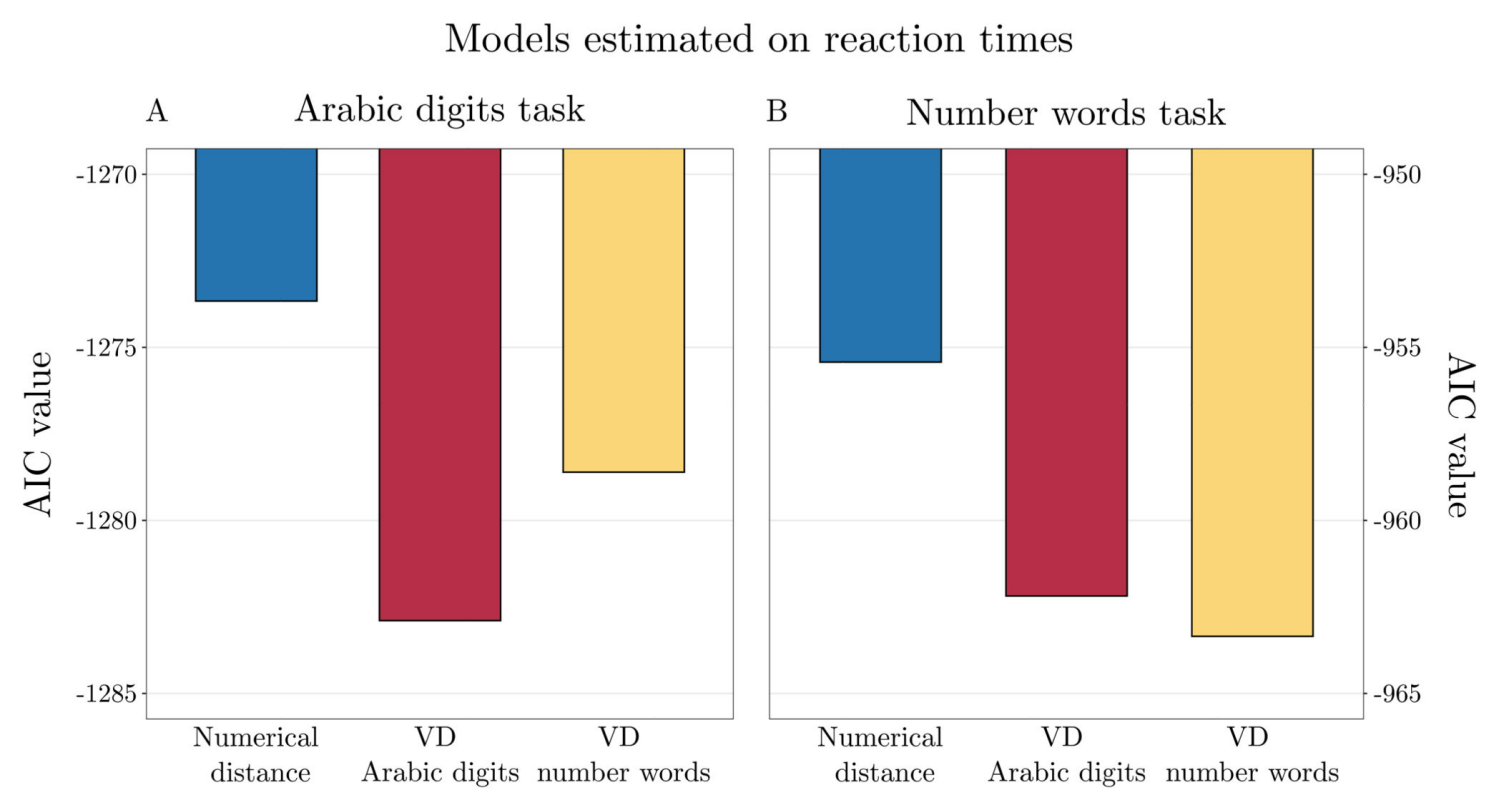
AICs of the models estimated on reaction times in the Arabic digits task (A) and in the number words task (B). In the Arabic digits task (A), the model including Arabic digits VD outperformed the other two models, indicating a notation-dependent effect. In the number words task (B), both VD models outperformed the numerical distance model. However, the number words VD and Arabic digits VD models were equally good at predicting participants’ chronometric performance.

In the Arabic digits task, all the predictors showed significant effects on RTs. To compare model fit, we calculated AIC values for all the models tested (i.e., without applying model criticism). The AIC values were as follows: real numerical distance model, AIC = −1273.662; Arabic digits VD AIC = −1282.894; and number words VD model, AIC = −1278.606. Therefore, according to Akaike weights, the model including Arabic digits VD outperformed the two other models by ΔAIC = 9.232 and ΔAIC = 4.288 respectively. This pattern of results is indicative of a notation-dependence effect (Fig. 4A).

#### 4.1.3 Number words task

In the number word task, we found a significant negative effect of numerical distance on RTs, *t*(19.0088) = −4.518, *p* < .001 (see Fig. 3D). Similarly, a significant negative effect of VD derived from Arabic digits, *t* (19.00391) = −4.498, *p* < .001 (see Fig. 3E) was also found, as well as for VD derived from number words, *t*(19.01452) = −5.017, *p* < .*001* (see Fig. 3F).

#### 4.1.4 Models comparison in the number words task

In the number words task, all the predictors showed significant effects on RTs. To compare model fit, we calculated AIC values for all the models tested (i.e., without applying model criticism). The AIC values were as follows: numerical distance model, AIC = −955.426; Arabic digits VD model, AIC = −962.183; and number words VD model, AIC = −963.349. Based on Akaike weights, both number words model (ΔAIC = 7.923), and Arabic digits based model (ΔAIC = 6.756) outperformed the numerical distance model. In the comparison of number words VDs and Arabic digits VDs based models, the ΔAIC = 1.166 indicates that both are equally good at predicting the participants’ chronometric performance in this task. These results are not in line with a notation-dependent view of numerical representation (Fig. 4B).

### 4.2 Accuracy

#### 4.2.1 Arabic digits task

For accuracy data, in the Arabic digits task, we found a significant positive effect of numerical distance, *z* = 3.956, *p* < .001. A significant positive effect was found also for the VD derived from Arabic digits, *z* = 3.607, *p* < .001, as well as for the VD derived from number words, *z* = 3.549 *p* < .*001*.

#### 4.2.2 Models comparison for accuracy in the Arabic digits task

**Fig. 5:**
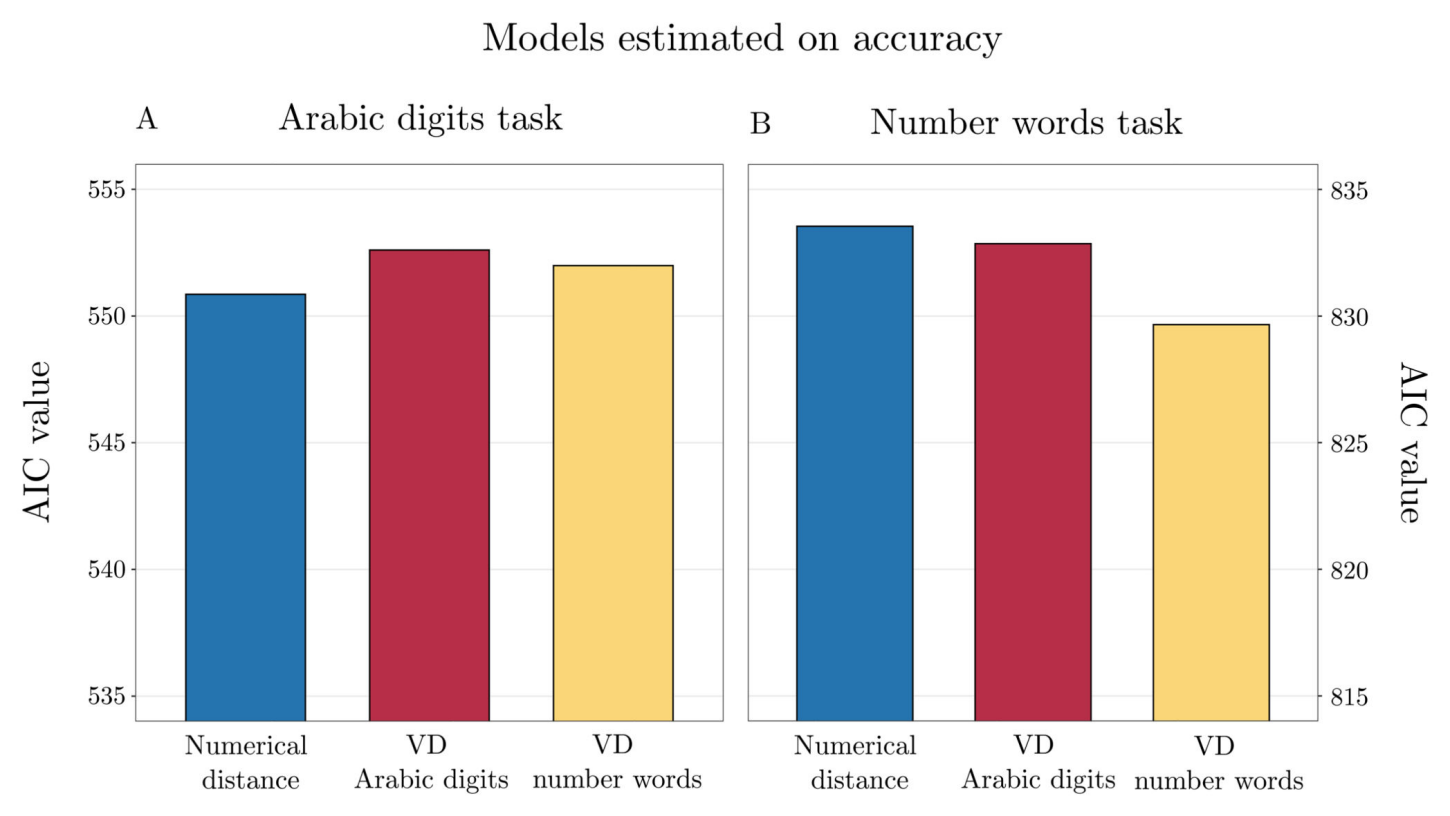
AICs of the models estimated on accuracy in the Arabic digits task (A) and in the number words task (B). In the Arabic digits task (A), the three models were equally good at predicting participants’ accuracy. In the number words task (B), the number words VD model outperformed the other two models, indicating a notation-dependent effect.

In the Arabic digits task, all the predictors showed significant effects on accuracy. To compare model fit, we calculated AIC values for all the models tested (i.e., without applying model criticism). The model based on real numerical distance had AIC = 550.8585, the model with Arabic digits VD had AIC = 552.6039, and the model with number words VD had AIC = 551.9913. Hence, there was ΔAIC = 1.745 between Arabic digits VD model and numerical distance model, ΔAIC = 1.133 between number words VD and numerical distance model, and ΔAIC = 0.613 between both VD based models. With the differences of AIC values <2, we interpret them as equally good at predicting participants’ accuracy. These results are not in line with a notation-dependent view of numerical representation (Fig. 5A).

#### 4.2.3 Number words task

For accuracy data, in the number words task, we did not find a significant effect of numerical distance, *z* = 1.609, *p* = .108. Likewise, there was no significant effect of VD derived from Arabic digits; *z* = 1.786, *p* = .074. Crucially, we found a significant positive effect of VD derived from number words; *z* = 2.494, *p* = .013. These results indicate that accuracy in the number words task is significantly predicted only by linguistic data from the respective notation.

#### 4.2.4 Models comparison for accuracy in the number words task

The model based on real numerical distance had AIC = 833.5460, the model with Arabic digits VD had AIC = 832.8575, and the model with number words VD AIC = 829.6616. Considering the fact that only number words VD model reached significance threshold, and the difference from the two other models is bigger than AIC = 2, we interpret it as the best in predicting participants’ accuracy. This pattern of results is indicative of a notation-dependence effect (Fig. 5B).

## 5 Discussion

In the present work, we investigated the differences and similarities between two numerical notations – Arabic digits and number words – by taking advantage of computational linguistics. Our aim was to quantify their relative distributional pattern in language and to predict behavioral performance in number comparison tasks using these experiential priors. In particular, the DSM used here was *fastText* (Joulin et al., 2016), a prediction-based model relying on cognitively-plausible, associative learning mechanisms (Mandera et al., 2017; Günther et al., 2019).

In Experiment 1, we observed that linguistic proxies for distance, as derived from DSM, for both Arabic digits and number words reliably capture the real numerical distance. In the behavioral experiment (i.e., Experiment 2), results showed faster RTs in the Arabic digit task as compared to the number word task. This appears in line with the *encoding complex hypothesis* (Campbell, 1994) which accounts for notation-dependent behavioral differences in numerical processes. However, this result may also be simply explained by the difference in string length, which is shorter for Arabic digits.

Participants’ chronometric performance was better accounted for by the linguistic indices (i.e., VDs) as compared to real numerical distance across both tasks. Critically, in the Arabic digits task, we found only a partial notation-dependent pattern of results, with linguistic predictors calculated from Arabic digits data constituting a better fit in accounting for chronometric performance. In contrast, model comparison for accuracy data in the Arabic digit task suggested no crucial difference between the predictors in explaining participants’ performance. For the number words task, we again found only a partial notation-dependent pattern of results. Analysis of reaction times showed that linguistic predictors from both notations are equally good at explaining participants’ performance. However, when considering accuracy data, we found that only the linguistic index derived from number words significantly predicted participants’ performance.

Thus, to summarize, our results revealed a partial notation-dependent pattern. In the Arabic digits task, notation dependence was observed for chronometric performance but not for accuracy, whereas in the number words task, it was observed for accuracy but not for chronometric performance. This dissociation may reflect differences in processing demands: Arabic digits, being more frequently used for quantification, might allow for faster access to magnitude representations, leading to linguistic influence at first stages of processing. In contrast, number words, which are less routinely used for quantification, might require more controlled and error-prone decision-making, making participants more sensitive to the specific linguistic experiential prior at the accuracy level.

The observation that the distributional history of numbers in language not only mirrors a distance effect-like relationship for both numerical notations but also predicts behavioral performance in numerical task replicates and extends previous evidence (Rinaldi et al., 2022), with novel findings about Arabic digits. Indeed, Rinaldi et al., 2022 reported a task-dependent dissociation: linguistic experiential priors better explained performance in symbolic tasks involving number words, whereas numerical ratio was a stronger predictor for non-symbolic tasks (i.e., dot arrays). Building on this evidence, here we strengthen this view at a finer level within symbolic processing, by assessing participants’ performance in comparison tasks employing both number words and Arabic digits. In doing so, we showed that linguistic experiential priors not only provide a better account of performance in symbolic comparison tasks than numerical distance, but we additionally found evidence for (partial) notation-dependent behavior. Notably, previous research suggested that participants’ performance with dot arrays and number words but not with Arabic digits could be predicted by linguistic priors (Ren & Libertus, 2024). In contrast, our results suggest that comparative judgments of Arabic digits can be predicted by linguistic priors. This discrepancy in results could be explained in light of the different DSMs used. Indeed, Ren and Libertus (2024) employed a traditional count-based model, which may struggle with tokens characterized by rare co-occurrences and poor contextual information (Mahmoud & Zrigui, 2021; Mandera et al., 2017). Alternatively, divergent results may also stem from the size of the linguistic corpora used across these studies: the TASA corpus employed by Ren and Libertus (2024) contains approximately 10 million words (Günther, Dudschig, & Kaup, 2015; Kanerva, Kristoferson, & Holst, 2000), while the version of *fastText* used here is trained on approximately 11 billion words (Joulin et al., 2016).

In addition to this, Ren and Libertus (2024) found that participants’ performance in the comparison tasks with number words was better predicted by the real ratio between the to be compared numbers than by the cosine similarity of vectors derived from LSA. This contrasts with our results, as well as those reported by Rinaldi et al. (2022) on a symbolic task. Both here and in Rinaldi et al. (2022) participants’ reaction times were better accounted for by: (i) the linguistic indices compared to numerical distance in the present study, and (ii) by linguistic estimates compared to numerical ratio in Rinaldi et al. (2022). Please note that linguistic estimates used by Rinaldi et al. (2022) encompassed both distance- and size-related proxies. On the other hand, the predictors used by Ren and Libertus (2024), namely numerical ratio and cosine similarity, are neither mathematically nor theoretically equivalent. The real numerical ratio indeed incorporates both the distance and size of the numbers; in contrast, cosine similarity only accounts for vector distance.^3^

Taken together, our findings, alongside the partial notation sensitivity exhibited by specific predictors, align with the information theory perspective and its core principle of efficient coding (Shannon, 1948). This mathematical framework underscores the optimization of neural responses based on the frequency of stimuli in the environment, positing heightened precision in perception for more frequently occurring stimuli (Atick & Redlich, 1992; Attneave, 1954). While traditionally applied to the neuroscientific study of perceptual systems (see Loh & Bartulovic, 2014), the learning process from language is ideally suited to be guided by these very same principles. Indeed, our results indicate that the (subtle) differences in patterns of usage for number words and digits may reflect the way we represent them. That is, the fact that the distribution of these two notations is highly correlated and yet these extremely nuanced differences between them can partially account for the observed discrepancy in behavior, provide some support to our theoretical claims. The notation-dependent behavioral and consequently representational disparity can be therefore interpreted as an effect of these slightly different contexts in which we encounter and learn number words and digits reflected by the DSM data. Findings support the idea that the language domain is likely to be governed by the same principles of efficient coding postulated for sensory processing in information theory interpretations (Loh & Bartulovic, 2014). Hence, the similarities and differences observed in the processing of different numerical notations might be harked back to the perceived notation-dependent regularities in which these particular notations occur and that facilitate statistical learning.

An interesting open question concerns the developmental trajectory of these effects. While some studies suggest that infants are sensitive to numerosity very early in life (e.g., Xu & Spelke, 2000; Jordan & Baker, 2011; but cf. Cantrell & Smith, 2013), comparison abilities progressively increase throughout development (Halberda & Feigenson, 2008; Mazzocco, Feigenson, & Halberda, 2011). Alongside, children’s exposure to language progressively accumulates, raising the possibility that the contribution of linguistic experiential priors strengthens with age. Future research could test this hypothesis by applying the present approach to younger populations, tracking the developmental emergence of linguistic effects, and examining whether its trajectory differs between digits and number words. In addition, complementary approaches using recent developments in language models designed to capture children’s linguistic experience (e.g., Ghanizadeh & Dousti, 2025) could provide further insights, as these models simulate language acquisition from limited, child-directed input and allow for systematic manipulation of linguistic experience.

Results of our behavioral experiment also present interesting implications for the longstanding symbol grounding issue of numerical processing, which relates to the question of how symbols acquire a numerical meaning (Harnad, 1990; Leibovich & Ansari, 2016). The traditional account states that the symbolic faculty for numbers is mapped onto an evolutionary old ANS designed to process all varieties of nonsymbolic magnitudes (e.g., physical size, weight, number of objects, etc.) (Cantlon et al., 2009; Dehaene, 1997). According to this perspective, humans and other species are born with an automatic ability to process nonsymbolic numerosities (Agrillo, Piffer, & Bisazza, 2011; Libertus & Brannon, 2009; Nieder, 2005). Symbolic representations for numerosities, such as Arabic digits or number words, would be learned later in development by mapping into these pre-existing nonsybolic representations. Hence, the ANS has been considered an ancient adaptation that came into being far before language and that ultimately governs numerical processes in both symbolic and non-symbolic format. A common argumentation for this account points to the presence of equivalent behavioral effects (e.g., ratio, size, and distance effects) in both symbolic and non-symbolic numerical tasks (Halberda, Ly, Wilmer, Naiman, & Germine, 2012; Moyer & Landauer, 1967; Xu & Spelke, 2000).

While observed similarities between numerical notations seemingly favor the theory of an ANS overlap in representation, opposite evidence and theoretical accounts are also present (for reviews see: Leibovich & Ansari, 2016; Reynvoet & Sasanguie, 2016). For example, by using computational modeling, Verguts and colleagues (2005) provided an explanation for the distance effects as emerging from network properties without the need of overlapping representations. Moreover, it has been shown that the size effect in symbolic comparisons is a consequence of a skewed frequency distribution of numbers (i.e., small numbers are more frequent than larger ones). Indeed, the size effect does not emerge with model simulations testing non-skewed distribution (Verguts & Van Opstal, 2014). Importantly, evidence against the ANS account has been reported also at the behavioral level when tasks other than the commonly used comparison task are used (e.g., Lyons, Ansari, & Beilock, 2012; Sasanguie, De Smedt, & Reynvoet, 2017).

Adding to this, by using DSM-based measures, we observed differences between Arabic digits and number words that were possible to model via their respective linguistic predictors. Therefore, it may be argued that there is no need to propose the explanation for the observed behavioral effects as relying solely on ANS.

These conclusions – which leverage natural language as a learning environment – share conceptual similarities with the Discrete Semantic Systems (DSS) model (Krajcsi et al., 2016, 2018), as both approaches assume that symbolic numerical processing is shaped by the statistical regularities encountered in experience, particularly emphasizing associative learning mechanisms. In both frameworks, the behavioral distance effect can emerge as a byproduct of these learned regularities, without requiring a single abstract magnitude representation. However, the two lines of evidence differ in the type and temporal scale of statistics they emphasize. Our results emerge from the distributional patterns of number symbols in natural language, to which individuals are continuously exposed, whereas evidence within the DSS perspective highlights more contextual environmental statistics arising within the experimental context. For instance, in the study by Krajcsi and Kojouharova (2017), after learning mappings between numerical values and novel arbitrary symbols, participants’ comparison performance was strongly shaped by recently acquired statistical information rather than by canonical values, thus contrasting ANS predictions.

Taken together, these perspectives suggest that long-term linguistic exposure and short-term contextual statistics likely operate jointly in shaping symbolic numerical representations (see also Boroditsky, 2001). More broadly, this interpretation is consistent with theoretical proposals suggesting that conceptual knowledge is shaped by multiple experiential sources, whose relative influence varies as a function of both long-lasting and contextual demands (Anceresi et al., 2025; Kemmerer, 2015).

Overall, our findings partially align with the studies in numerical research pointing at notation dependency in the processing of symbolic numbers (Kadosh et al., 2008), and adds to revious studies with non-symbolic and symbolic numbers comparison (Rinaldi et al., 2022; Ren & Libertus, 2024), providing a plausible explanation for differences observed in number processing of various number presentations (Krajcsi et al., 2018). Our study stands in contrast to those theoretical accounts postulating a single, notation-independent representation for all the numerical processing (Dehaene & Mehler, 1992). It also adds to building a body of evidence pointing at a wide range of uses for DSMs, with the first evidence reported here – to our knowledge – showing the discrepancy between two notations for numbers with distributional semantics models.

## Data Availability

Supplementary materials related to this article, including the stimuli, generated vector spaces, datasets, and analysis scripts, are openly available on the Open Science Framework (https://osf.io/geq5y/).

## Footnotes

2 Computed through the Hittner, May, and Silver’s (2003) modification of Dunn and Clark’s z (1969), using a backtransformed average Fisher’s (1921) Z procedure.

3 Importantly, although previous studies have proposed a distributional operationalization of a size-like effect (i.e., Vector Variance, VV; see Rinaldi & Marelli, 2020; Rinaldi et al., 2022), identifying a comparable linguistic analogue within the present modeling framework is less straightforward. The DSM used here incorporates subword information (Bojanowski et al., 2017), which improves the robustness of semantic representations for measuring vector distance (i.e., a proxy for the distance effect) but makes VV less reliable as an index of contextual predictability because it is influenced by shared orthographic *n*-grams. For this reason, the present work specifically addresses the distance effect in numerical comparison.
